# Serotonin Application Decreases Fluoxetine-Induced Stress in *Lemna minor* and *Spirodela polyrhiza*

**DOI:** 10.3390/ijms27010002

**Published:** 2025-12-19

**Authors:** Marta Wierzbicka, Dariusz J. Michalczyk, Agnieszka I. Piotrowicz-Cieślak

**Affiliations:** Department of Plant Physiology, Genetics and Biotechnology, University of Warmia and Mazury in Olsztyn, 10-720 Olsztyn, Poland; marta.wierzbicka@student.uwm.edu.pl (M.W.); darim@uwm.edu.pl (D.J.M.)

**Keywords:** selective serotonin reuptake inhibitors, toxicity, duckweeds, oxidative stress, heat-shock proteins, phytoremediation

## Abstract

The aim of this study was to evaluate the impact of fluoxetine, a widely used selective serotonin reuptake inhibitor, on two aquatic plants: *Lemna minor* and *Spirodela polyrhiza*. Additionally, the effect of exogenous serotonin on the level of fluoxetine-induced stress in duckweed will be studied. Increasing presence of antidepressants in surface waters poses ecological risks, and the duckweed species are ideal model organisms for ecotoxicological studies due to their rapid growth and ability to accumulate pollutants. For 14 days, plants were exposed to fluoxetine (0.001–150 mg L^−1^), followed by a recovery phase in a drug-free medium or a medium supplemented with exogenous serotonin. We analysed morphological/physiological parameters (frond length and area, fresh and dry mass, hydration, stomatal size), the activity of antioxidant enzymes (catalase, ascorbate peroxidase, superoxide dismutase), cell viability, and the level of heat-shock proteins. The plants’ ability to remove fluoxetine from the medium was also assessed. High fluoxetine concentrations (50–150 mg L^−1^) significantly reduced fresh mass (by 63–98% in *L. minor* and 56–97% in *S. polyrhiza*), frond area (by 21–48% in *L. minor* and 11–25% in *S. polyrhiza*), and cell viability (by 36–94% in *L. minor* and 49–94% in *S. polyrhiza*), and induced oxidative stress. Despite this, both species showed high regeneration potential after the stressor’s removal. Serotonin supplementation did not affect morphology but increased antioxidant enzyme activity, improved cell viability, and elevated heat-shock proteins levels. Crucially, serotonin significantly increased the efficiency of fluoxetine removal. The data can provide a basis for predicting fluoxetine removal efficiency in plants with different levels of endogenous serotonin. *L. minor* and *S. polyrhiza* exhibit substantial tolerance to fluoxetine, and antioxidative enzymes are sensitive markers of this stress.

## 1. Introduction

*Lemna minor* L. and *Spirodela polyrhiza* (L.) Schleid., commonly known as duckweeds, are small pleustonic plants with a cosmopolitan distribution, especially across the temperate Northern Hemisphere [1]. They are often used as bioindicators of water quality and as model species in ecotoxicological studies [2]. Duckweeds are also applied in phytoremediation, owing to their ability to accumulate nutrients and contaminants from aquatic environments, including heavy metals, pesticides, and pharmaceuticals [3].

The high consumption of antidepressants raises environmental concerns, as these compounds, along with other personal care products (PPCPs) and endocrine-disrupting chemicals (EDCs), are detected in wastewater and surface waters with increasing frequency [4].

In recent years, antidepressants, particularly selective serotonin reuptake inhibitors (SSRIs), have emerged as significant water contaminants. The volume of sales of these drugs showed an average annual growth of 4% between the year 2008 and 2019. The use of psychiatric drugs in Brazil in this period increased by 220%, in Poland by nearly 60%, in Australia by 45%, and in Canada by almost 28% [5]. During the COVID-19 pandemic, and even after its resolution, anxiety triggered by the virus led to a notable additional increase in antidepressant consumption in some countries. An inquiry carried out in Europe revealed that in 2023 almost every other respondent (46%) experienced emotional and psychological problems like depression or anxiety [6]. Antidepressants are commonly used to treat such problems [7]. Fluoxetine, citalopram, and sertraline represent key SSRIs, with fluoxetine being the most widely prescribed since the 1980s [5].

World average concentrations of fluoxetine in surface waters range from 12 to 1400 ng g^−1^ [8,9,10]. Fluoxetine was also found to accumulate in plants, reflecting its persistence and bioavailability [11].

Serotonin (Ser), on the other hand, is the target molecule of SSRI in animals, and a well-known neurotransmitter that regulates mood, sleep, anxiety, and various physiological processes. However, it occurs naturally in some plants too, including species such as *Juglans regia* and *Carya* spp. In plants, Ser was first identified in the legume *Mucuna pruriens* [12] and later found in approximately 42 plant species from 20 families [13]. It occurs in leaves, stems, roots, fruits, and seeds. Its roles in plants include contribution to the regulation of growth, flowering, and stress responses [14]. The Ser concentrations in plants show a considerable variation, ranging from 3 to 30 mg kg^−1^, but occasionally reaching as much as 400 mg kg^−1^ [15]. Ser has also been detected in the aquatic plant *Lemna aequinoctialis* [16].

The aim of this study was to evaluate the effect of fluoxetine on the morphological, physiological, and biochemical parameters of two aquatic plant species: *L. minor* and *S. polyrhiza*. Fluoxetine is one of the most widely used antidepressant drugs, belonging to the SSRI class. We assessed whether exogenous Ser modulates the plant’s response to fluoxetine-induced stress and assists in plant regeneration following exposure to the drug. Furthermore, the study determined the plants’ ability to remove fluoxetine from the medium and the influence of Ser on this process.

## 2. Results and Discussion

We investigated the effect of fluoxetine and Ser in the medium on plant growth and physiological–biochemical parameters as well as the recovery of *L. minor* and *S. polyrhiz*a after the drug’s removal. Therefore, we carried out two experiments: A. Plant responses to fluoxetine and serotonin applied separately; B. Plant responses to a combined application of fluoxetine and serotonin.

We also analyzed the effect of Ser added after the fluoxetine treatment on plant recovery. The plants were cultivated for 14 days in a medium containing fluoxetine (at concentrations from 0.001 to 150 mg L^−1^ and then for another 14 days without fluoxetine (0 Flu) and with the addition of 100 µM Ser (0 Flu + Ser). The regeneration capacity was assessed by analyzing physiological and biochemical parameters, including the surface area and length of transformed fronds; stomatal length; dry and fresh weight and hydration; antioxidant enzyme activity; cell viability; and heat-shock proteins—HSP70 content.

### 2.1. Plant Responses to Fluoxetine and Serotonin Applied Separately

#### 2.1.1. Morphological Parameters

**Figure 1:**
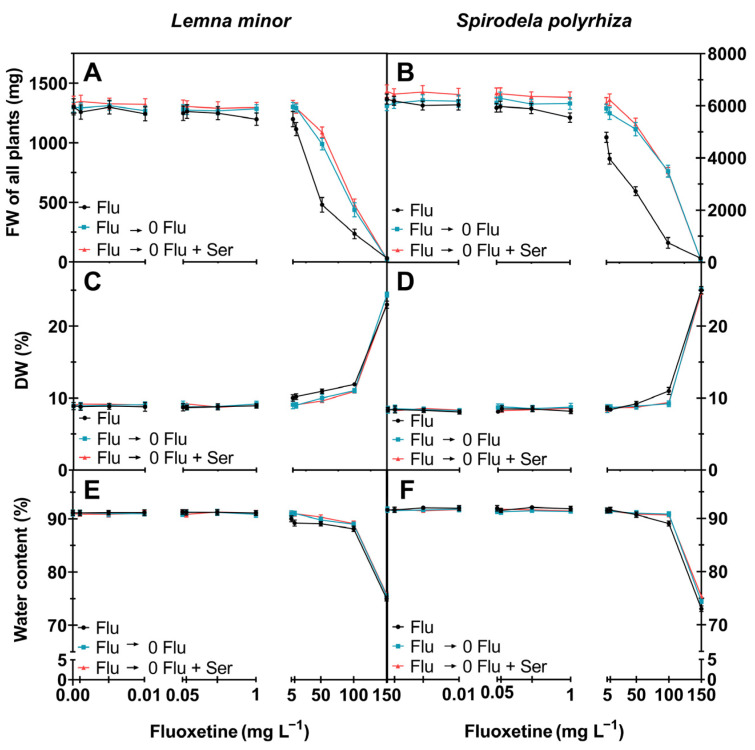
Morphological parameters of *L. minor* and *S. polyrhiza* in fluoxetine concentrations (0; 0.001; 0.005; 0.01; 0.05; 0.1; 0.5; 1; 5; 10; 50; 100; 150 mg L^−1^): (**A**,**B**)—shoot weight (mg); (**C**,**D**)—dry weight (%); (**E**,**F**)—water content (%). Mean ± SD (standard deviation), *n* = 3. Flu (**―**)—fluoxetine exposure before recovery; Flu ⟶ 0 Flu (**―**)—recovery; Flu ⟶ 0 Flu + Ser (**―**)—recovery with 100 µM Ser. Statistical analyses and details are provided in Supplementary Tables S1–S6.

Our results clearly indicate that the fresh weight of *S. polyrhiza* significantly decreased at fluoxetine concentrations of 5, 10, 50, 100, and 150 mg L^−1^, by 23, 37, 56, 88, and 97% compared to control (Figure 1B). A more-than-twofold increase in percent dry weight was observed in plants treated with the highest fluoxetine concentration of 150 mg L^−1^ (Figure 1C,D). This change reflected the plants’ water loss, with hydration decreasing by 20% at the highest fluoxetine concentration used (Figure 1E,F).

**Figure 2:**
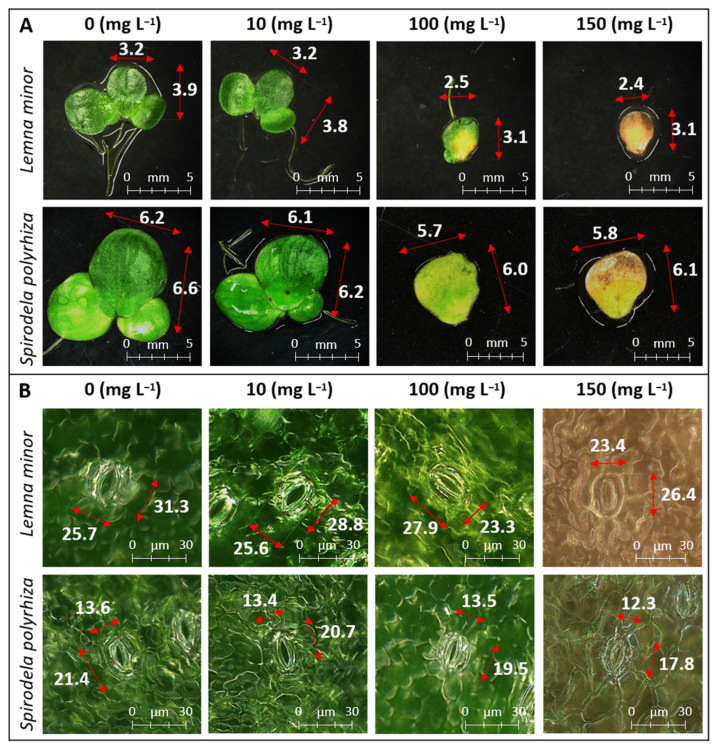
*L. minor* and *S. polyrhiza*: (**A**)—frond length and width (mm); (**B**)—stomatal apparatus length and width (µm); measured in control and after exposure to 10, 100, and 150 mg L^−1^ fluoxetine.

Along with the sharp changes in fresh and dry weight and the decrease in plant hydration at the highest fluoxetine concentration, plant chlorosis was also observed (Figure 2A).

**Figure 3:**
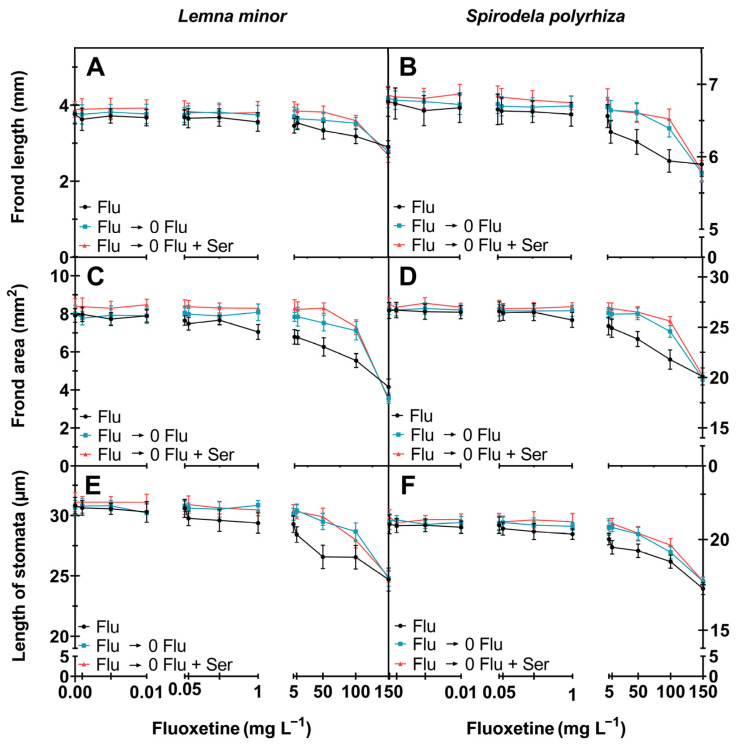
Morphological parameters of *L. minor* and *S. polyrhiza* determined in fluoxetine concentrations (0; 0.001; 0.005; 0.01; 0.05; 0.1; 0.5; 1; 5; 10; 50; 100; 150 mg L^−1^): (**A**,**B**)—frond length (mm); (**C**,**D**)—frond area (mm^2^); (**E**,**F**)—stomatal apparatus length (µm). Mean ± SD, *n* = 30. Flu (**―**)—fluoxetine exposure before recovery; Flu ⟶ 0 Flu (**―**)—recovery; Flu ⟶ 0 Flu + Ser (**―**)—recovery with 100 µM Ser. Statistical analyses and details are provided in Supplementary Tables S7–S12.

The frond length of *S. polyrhiza* and *L. minor* decreased at very high concentrations of fluoxetine (50–150 mg L^−1^; Figure 3A,B). However, two other morphological parameters appear to be more sensitive to fluoxetine’s detrimental effects than the frond length: the frond surface area and the stomatal length (Figure 2). The decrease in frond surface area was particularly visible in *L. minor* cultured in fluoxetine-containing medium (with the fluoxetine concentration of 150 mg L^−1^. Under these conditions a more than 47% decrease in frond surface area was observed when compared to the control (Figure 3C). Significant decreases in the frond surface area of *S. polyrhiza* were also noted at concentrations of 100 and 150 mg L^−1^ (by 18 and 25%, respectively). During the recovery phase, the stomatal length of the plants increased and reached values comparable to the control, even after exposure to a fluoxetine concentration of 50 mg L^−1^ (Figure 3E,F).

Analysis of the growth parameters showed that both *L. minor* and *S. polyrhiza* have a high regenerative potential, i.e., frond area of *L. minor* increased by 20%, and that of *S. polyrhiza* by over 10% during the recovery phase- after exposure to 50 mg L^−1^ fluoxetine and subsequent transfer to a fluoxetine-free medium (0 Flu). With the highest concentration of fluoxetine, however, no regeneration was observed. The fresh weight of *L. minor* plants, first exposed to fluoxetine (50 mg L^−1^) and then transferred to fluoxetine-free medium (both with and without Ser addition), more than doubled in comparison to the plants constantly exposed to fluoxetine. The fresh weight of *S. polyrhiza* subjected to analogous treatment also increased its fresh mass almost twice in both variants of the recovery phase (relative to continuous exposure). Similar results were obtained by Krupka et al. [17], who treated *L. minor* with tetracycline. These authors showed that *L. minor* was able to regenerate after the stressor was removed, and all growth parameters, including plant count, leaf area, and fresh and dry weight, increased by about 40% after the plants were transferred to a tetracycline-free medium. The addition of Ser, tested in the current study during the recovery phase, did not cause significant changes in the morphological parameters of the plants examined; however, it did improve some physiological (frond area) and biochemical (catalase (CAT), ascorbate peroxidase (APX), superoxide dismutase (SOD) activity, cell viability, and heat-shock protein content) parameters. The role of exogenous Ser in aquatic plants remains poorly understood, although emerging evidence indicates that this compound may exert hormone-like functions. In animals, Ser is a well-characterized neurotransmitter involved in the regulation of mood, circadian rhythms, and stress responses. In plants, its occurrence has been documented in multiple terrestrial species as well as in the aquatic plant *L. aequinoctialis* [16], suggesting that its presence is widespread. Ser is implicated in processes central to plant growth and development, including root system regulation, shoot organogenesis, biomass accumulation, and responses to abiotic and biotic stress [14].

In addition to the morphological indices, the EC_50_ (Effective Concentration 50%) value for the growth rate, using the number of *L. minor* and *S. polyrhiza* plants, was determined. The value of this indicator for *L. minor* was fluoxetine 31.6 ± 5.2 mg L^−1^, and for *S. polyrhiza* it was fluoxetine 21.5 ± 3.8 mg L^−1^. A similar level of EC50 (27.0 ± 8.7 mg L^−1^) for fluoxetine toxicity to *L. minor* was obtained by Ramirez-Morales et al. [18]. Other publications indicate that the fluoxetine concentration causing a 50% decrease in the number of *L. minor* fronds is 8.04 ± 0.40 mg L^−1^ and 6.04 mg L^−1^ [19,20]. Brain et al. [21] did not show any significant changes in plant numbers at fluoxetine concentration of 1 mg L^−1^. In the literature, EC_50_ values for *L. minor* growth have rarely been analyzed, and the results obtained are inconsistent [17]. These discrepances suggest that the use of EC_50_ as a sole measure of phytotoxicity may be problematic.

Another group of commonly used model organisms in ecotoxicological studies are green algae [22]. Silva et al. [23] found that fluoxetine concentrations in the range of 0.25 to 15 mg caused a 50% growth inhibition of the alga *Chlorella vulgaris* (these results were obtained for three different formulations/brands of the drug).

#### 2.1.2. Enzyme Activity

Reactive Oxygen Species (ROS) are important for plant growth and development and participate in various signaling cascades that are responsible for providing defense against abiotic and biotic stresses [24]. In plants, ROS are free radicals, such as the superoxide anion, hydroxyl radical, hydroperoxyl radical, and alkoxyl radical, as well as non-radical compounds like hydrogen peroxide and singlet oxygen [25]. Similarly, Reactive Nitrogen Species (RNS) is a collective term that includes free radicals, such as nitric oxide and nitrogen dioxide, and non-radicals, such as peroxynitrite and the nitroxyl anion [26]. Both ROS and RNS are a consequence of aerobic metabolism [27]. Fundamental discoveries in ROS/RNS research have confirmed a complex signaling network linking them to a wide range of biological processes in all living organisms [27,28]. However, the uncontrolled accumulation of ROS is highly toxic to the cell [29]. Antioxidant enzymes responsible for removing ROS include SOD, APX, and CAT [30].

**Figure 4:**
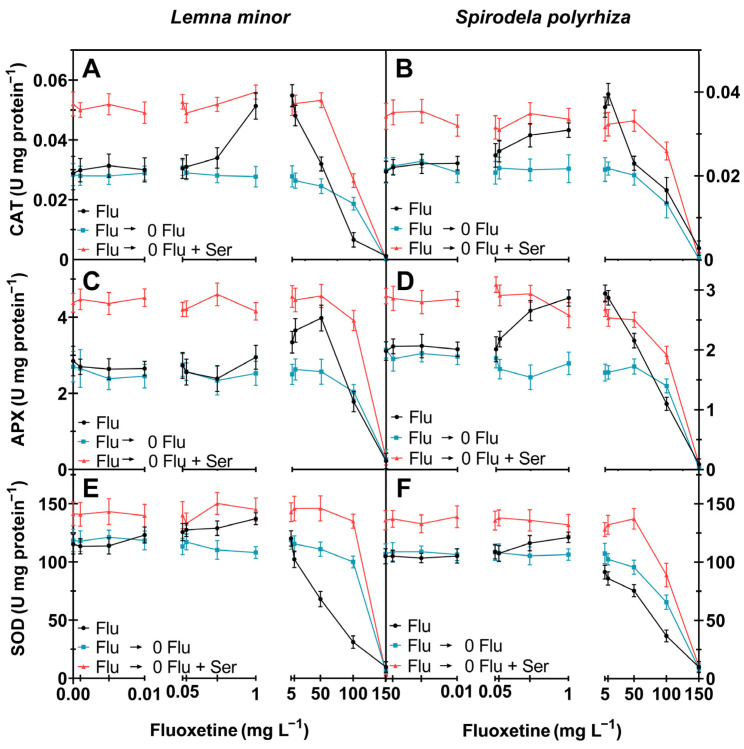
Biochemical parameters of *L. minor and S. polyrhiza* determined in fluoxetine concentrations (0; 0.001; 0.005; 0.01; 0.05; 0.1; 0.5; 1; 5; 10; 50; 100; 150 mg L^−1^): (**A**,**B**)—Catalase activity (U mg protein^−1^); (**C**,**D**)—Peroxidase activity (U mg protein^−1^); (**E**,**F**)—Superoxide dismutase activity (U mg protein^−1^). Mean ± SD, *n* = 3. Flu (**―**)—fluoxetine exposure before recovery; Flu ⟶ 0 Flu (**―**)—recovery; Flu ⟶ 0 Flu + Ser (**―**)—recovery with 100 µM Ser. Statistical analyses and details are provided in Supplementary Tables S13–S18.

CAT (EC 1.11.1.6) is an enzyme that directly converts hydrogen peroxide into water and oxygen [31]. CAT activity in *L. minor* was nearly 78% and 90% higher at fluoxetine concentrations of 1 and 5 mg L^−1^, respectively, compared to the control (Figure 4A). At fluoxetine concentrations exceeding 10 mg L^−1^ a rapid decline in CAT activity in *Lemna* was observed. Similarly, the activity of this enzyme in *S. polyrhiza* reached its highest value at a fluoxetine concentration of 10 mg L^−1^, increasing by 88% (Figure 4B). This non-linear pattern of changes in CAT activity (an initial increase followed by a decrease) was also recorded in *L. minor* responding to other stresses (e.g., increasing concentrations of cadmium [32]) and in *S. polyrhiza* under copper and mercury stress [33].

APX (EC 1.11.1.1) catalyzes the reduction of hydrogen peroxide to water using ascorbate as an electron donor under both stressful and normal conditions [34]. During fluoxetine exposure, the highest APX activity was recorded at a 50 mg L^−1^ fluoxetine concentration for *L. minor* and at a 5 mg L^−1^ fluoxetine concentration for *S. polyrhiza,* representing an increase of 40% and 49%, respectively, compared to the control (Figure 4C,D). At the highest concentrations of fluoxetine APX activity sharply dropped and no activity was detected at fluoxetine 150 mg L^−1^. Similar results were obtained by researchers who treated *Ulva lactuca* with fluoxetine. APX activity increased up to a certain point, after which a decrease was noted [35]. Similar dynamics of changes in APX activity were observed for *L. minor* treated with cadmium [32].

SOD (EC 1.15.1.1) converts superoxide anions into hydrogen peroxide. Changes in SOD activity in plants appear to be a much more sensitive parameter of fluoxetine’s harmfulness than other enzymes. The highest SOD activity for *L. minor* and *S. polyrhiza* was recorded at a drug concentration of 1 mg L^−1^ (Figure 4E,F). In both cases, there was a gradual decrease in SOD activity with increasing fluoxetine concentration. Similar results were obtained by researchers in a study on the effect of fluoxetine on *U. lactuca*. A decline in SOD activity was observed under fluoxetine concentrations of 40 and 80 µg L^−1^ [35]. Changes in enzyme activity can be caused by changes in gene expression. After exposure of the fungus *Trichophyton rubrum* to sertraline, a decrease in the expression of the gene encoding Fe-SOD (iron-dependent superoxide dismutase) was observed [36].

Ser acts as an antioxidant by scavenging ROS and exhibits strong in vitro antioxidant activity [37]. In the experiments described in the current paper, after the addition of Ser (0 Flu + Ser) during the plant recovery phase, the activity of all antioxidant enzymes in *L. minor* and *S. polyrhiza* was higher than without this addition (0 Flu) (Figure 3A–F). A similar effect (enzyme activity stimulation) was achieved by researchers analyzing the simultaneous application of salt stress and Ser to *Brassica napus*. Ser helps activate antioxidant defense systems, such as SOD, CAT, and APX [38]. These results are consistent with the conclusions of another study that investigated the effect of exogenous Ser on drought stress in *Crocus sativus* [39]. The effect of exogenously added Ser on the activity of CAT, APX, and SOD during the recovery phase of plants after the removal of a stress factor has not been studied before.

Transferring the plants to fluoxetine-free medium (0 Flu), within this study, mostly caused the activity of the antioxidant enzymes to return to values comparable to the control (Figure 3A–F). Only with the highest concentrations of fluoxetine no recovery to the control levels of enzyme activity. Drobniewska et al. [40] also examined the regenerative abilities of *L. minor* (treated with sulfadimethoxine), where a decrease in the activity of antioxidant enzymes was shown after the toxic agent was removed. On the other hand, in a similar study of the regenerative capacity of *L. minor* after exposure to tetracycline, an increase in the activity of antioxidant enzymes was shown after the toxic agent was removed [17].

#### 2.1.3. Cell Viability and Stress Response (HSP70)

**Figure 5:**
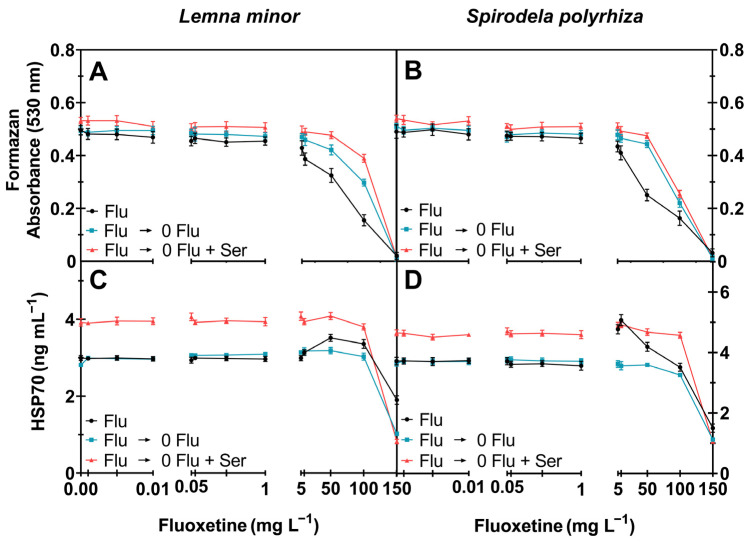
Biochemical parameters of *L. minor* and S. *polyrhiza* determined in fluoxetine concentrations (0; 0.001; 0.005; 0.01; 0.05; 0.1; 0.5; 1; 5; 10; 50; 100; 150 mg L^−1^): (**A**,**B**)—Viability test (TTC assay); (**C**,**D**)—HSP70 protein content (ng mL^−1^). Mean ± SD, *n* = 3. Flu (**―**)—fluoxetine exposure before recovery; Flu ⟶ 0 Flu (**―**)—recovery; Flu ⟶ 0 Flu + Ser (**―**)—recovery with 100 µM Ser. Statistical analyses and details are provided in Supplementary Tables S19–S22.

The TTC (2,3,5-triphenyltetrazolium chloride) assay is a common method for determination of plant tissue viability. This method is based on the enzymatic reduction of TTC to insoluble red formazan in metabolically active tissues [41]. Only living cells, which contain the mitochondrial dehydrogenase enzyme (which reduces TTC to red formazan), will be stained, while dead cells will not [42]. In the concentration range of 0 to 1 mg L^−1^, fluoxetine caused minor damage to oxidoreductase enzymes. The cell viability of *L. minor* significantly decreased when the fluoxetine concentration increased to 10 mg L^−1^ (by over 22%) (Figure 5A). A very large decrease in the cell viability of *S. polyrhiza* was recorded at a drug concentration of 50 mg L^−1^ (by over 48% compared to the control) (Figure 5B). Furthermore, a continued decrease in the cell viability of both plant species was noted with increasing fluoxetine concentration.

After transferring the plants to drug-free medium and medium with added Ser, we observed a significant increase in the total activity of dehydrogenases, particularly at fluoxetine concentrations from 5 to 100 mg L^−1^ for both studied aquatic plant species (Figure 5A,B).

Heat-shock proteins are expressed when stress occurs [43]. HSP70 exerts cytoprotective effects under various conditions, playing a key role in cellular protein quality control and degradation. It has been discovered that, in addition to their function of protecting cells against heat and oxidative stress, they serve as chaperone proteins involved in the process of protein folding. As their name suggests, heat shock proteins accumulate in the largest quantities during heat stress. They are also a biomarker of the stress response induced by anthropogenic factors. HSPs have been detected in chloroplasts, cell nuclei, cytoplasm, cell membranes, and mitochondrial membranes, among other places [44]. An increase in the amount of HSP70 was recorded in *L. minor* at fluoxetine concentrations of 50 and 100 mg L^−1^ (representing an 18% and 12% increase, respectively), while in *S. polyrhiza*, an increase was observed primarily at fluoxetine concentrations of 5 and 10 mg L^−1^ (representing a 28% and 36% increase, respectively); however, in both species there was a clear drop in HSP70 at the highest fluoxetine concentration—150 mg L^−1^ (Figure 5C,D). Similar results were obtained by Krupka et al. [17], who studied the effect of tetracycline on *L. minor*, and by Gorovits et al. [45], who studied the increase of HSP70 in tomato leaves exposed to drugs. On the other hand, Ziółkowska and Piotrowicz-Cieślak [46] recorded a decrease in the amount of HSP70 in *Pisum sativum* under the influence of sparfloxacin.

We observed an increased HSP70 content in both *L. minor* and *S. polyrhiza* (after exposure to fluoxetine concentrations of 0–100 mg L^−1^ during the recovery phase with added Ser (0 Flu + Ser), and values comparable to the control in plants regenerating without this addition (0 Flu) (Figure 5C,D). In contrast to these results, Krupka et al. [17] observed an increase in HSP70 content after a one-week recovery of *L. minor* from the stress induced by tetracycline.

The conducted research is of significant practical importance for both the ecology of surface waters and the development of phytoremediation for pharmaceutical-contaminated waters. Hypothetically, aquatic plants with high levels of endogenous Ser (including some duckweeds) may exhibit greater resistance to pharmaceutical contamination, which is crucial for maintaining the stability of aquatic ecosystems. Considering these data, future research should investigate the correlation between endogenous Ser levels and the efficacy of fluoxetine removal from the environment by plants. Duckweed species *(L. minor* and *S. polyrhiza*), due to their high capacity for regeneration after fluoxetine-induced stress, can be effectively utilized in water purification systems contaminated with this pharmaceutical, such as in constructed wetlands or phytoreactors.

### 2.2. Plant Responses to a Combined Application of Fluoxetine and Serotonin

#### 2.2.1. Fluorometric Determination of Fluoxetine Removal from the Medium by Plants

To improve the effectiveness of phytoremediation, it is recommended to use plants that produce a large amount of biomass, and to use chelating agents that increase the bioavailability of elements and improve plant tolerance to stress. One of the factors influencing the effectiveness of phytoremediation is the plant species used. Different BF (bioconcentration factor) values for heavy metals have been shown depending on the plant species studied. Sunflower bioaccumulates more cadmium and lead than sorghum or corn, but sorghum and corn can accumulate a larger amount of zinc in their tissues than sunflower [47]. Plants show varied substance distribution depending on the plant part. The effect of three antidepressant drugs on the plant *Lepidium sativum* was recorded in the literature. It was shown that its roots accumulated 4.29 ± 0.98 µg g^−1^ of sertraline, but more than four times less trazodone (0.91 ± 0.15 µg g^−1^). The opposite proportions/results were obtained when checking the bioaccumulation of substances in the plant’s leaves. The leaves of *Lepidium sativum* accumulated almost five times less sertraline (0.52 ± 0.14 µg g^−1^) than trazodone (2.63 ± 0.37 µg g^−1^) [48]. Plants can also increase their tolerance to a toxic agent. From first-generation *Solanum nigrum* plants grown in soil containing 50 mg kg^−1^ of cadmium (Cd), a plant form was obtained that showed increased resistance to Cd stress and a greater ability to bioaccumulate this element [49]. The effectiveness of phytoremediation also depends on the dose of the toxic agent. Exposing *L. minor* to increasingly higher fluoxetine concentrations caused a more intense removal of the drug from the medium (Supplementary Figure S1). It is known that *L. minor* and *S. polyrhiza* are used in the phytoremediation of environmental contaminants. They eliminate excess nitrogen and phosphorus from the substrate [50], antibiotics [51], and heavy metals [52,53].

During the exposure of *L. minor* to initial fluoxetine concentrations of 5, 10, 50, and 100 mg L^−1^, the percentage decrease in fluoxetine content in the medium was 0.5, 6, 19 and 31%, respectively (Table 1). A more pronounced fluoxetine removal was observed for the *S. polyrhiza* culture at low antidepressant concentrations, but less pronounced at high concentrations, with respective decreases of 3, 6, 14 and 14%. Similarly, Drobniewska et al. [19] showed that a higher fluoxetine concentration resulted in a twofold increase in the bioaccumulation of the drug by *L. minor*. Moreover, other studies on *Cucurbita pepo* by Carter et al. [54] showed that the uptake of carbamazepine and verapamil also increased in a concentration-dependent manner.

The addition of 100 µM Ser enhanced the removal of fluoxetine from the medium during our experiments. The percentage decrease in the initial fluoxetine level was 25, 63, 53, and 60% for *L. minor* and 29, 35, 37, and 43% for *S. polyrhiza* at initial fluoxetine concentrations of 5, 10, 50, and 100 mg L^−1^, respectively (Table 1). While the effect of exogenous Ser on the absorption of potentially toxic substances by plants has not yet been studied, the exogenous application of its precursor, melatonin, is known to modulate the bioaccumulation of various substances in plants. The addition of melatonin caused an increase in Cd accumulation within the roots and leaves of lemon balm (*Melissa officinalis*) [55]. Conversely, it was found that exogenous melatonin significantly lowered the Cd content in the roots and shoots of radish, suggesting that responses to melatonin may be species-specific. Furthermore, the same investigation revealed that melatonin enhanced the activity of antioxidant enzymes, thereby aiding the neutralization of Cd-induced oxidative stress [56].

#### 2.2.2. Stability of Fluoxetine and Serotonin in Solutions with No Plants

**Figure 6:**
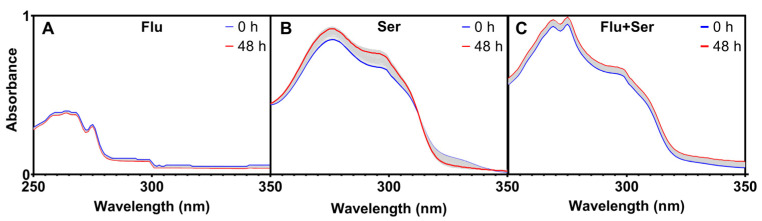
Absorbance changes (in the range of 250–350 nm): (**A**)—fluoxetine, (**B**)—serotonin, (**C**)—mixture of fluoxetine and serotonin (recorded every 2 min for 48 h at the temperature of 23 °C in darkness); 0 h—first measurement, 48 h—last measurement (after two days). A total of 1440 measurements were taken over 48 h for each variant.

Measurements were conducted to verify whether the decrease in fluoxetine concentration in the medium, observed in a previous experiment, was at least in part attributable to spontaneous degradation of the substance without plant involvement. To determine the intensity of photodegradation and thermal degradation, we investigated changes in the absorption spectra of the studied compounds over time and at different temperatures. In the dark, the absorption spectrum of fluoxetine decreased by only 4% over two days (at λ = 275 nm) (Figure 6A), which is consistent with the findings of Kwon and Armbrust [57], who reported a 2–3% degradation. In contrast, the absorbance of both serotonin and the fluoxetine–serotonin mixture increased by 8% and 12%, respectively (at λ = 275 nm) (Figure 6B,C).

**Figure 7:**
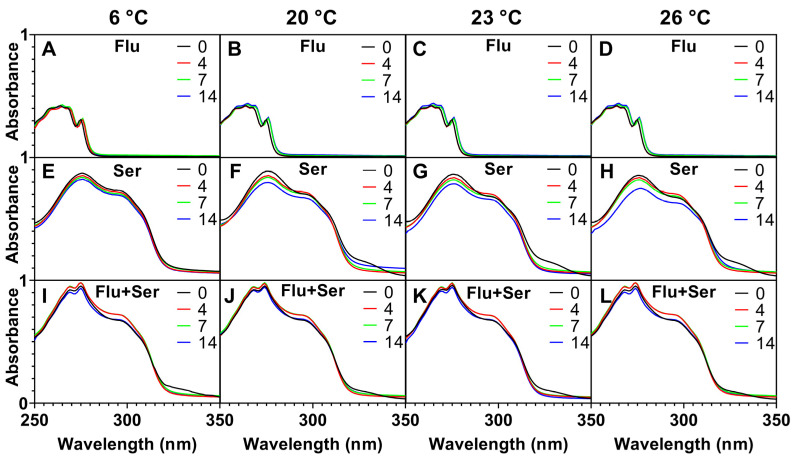
Absorbance changes (in the range of 250–350 nm): (**A**–**D**)—fluoxetine, (**E**–**H**)—serotonin, (**I**–**L**)—mixture of fluoxetine and serotonin; incubated for 0, 4, 7, 14 days; at temperatures of 6, 20, 23, and 26 °C; under a 16/8 h photoperiod.

Under a 16/8 h photoperiod, a gradual decrease in the absorbance of serotonin and the fluoxetine–serotonin mixture was recorded after 14 days, indicating their degradation (Figure 7E–L). The fluoxetine solution incubated at 6 °C with a 16/8 h photoperiod showed the highest stability over 14 days (Figure 7A). Fluoxetine was also not degraded at 23 °C (Figure S2). Based on these results, we assume that light and temperature did not significantly affect the removal of fluoxetine from the medium by phytoremediation.

The results unequivocally demonstrate that the decrease in fluoxetine concentration in the medium, observed in cultures of *Lemna* and *Spirodela* (c.f. Section 2.2.1), is indeed a result of the phytoremediation activity of the tested plants, rather than the spontaneous degradation of the substance.

Interestingly, the sensitivity of both plant species to fluoxetine was very similar. Moreover, the biomass of *Spirodela*, cultivated under both control conditions and in the presence of fluoxetine, was approximately four times greater than that of *L. minor*. Nevertheless, the amount of drug removed from the medium by *Lemna* was higher than that removed by *Spirodela*. When Ser was added to the medium, this advantage of *Lemna* over *Spirodela* was further enhanced, reaching 42% and 33% in *Lemna* and *Spirodela*, respectively.

## 3. Materials and Methods

### 3.1. Plant Material

The experimental material consisted of axenic plants of common duckweed (*Lemna minor* L.), and greater duckweed (*Spirodela polyrhiza* L.) obtained from the Department of Plant Physiology, Genetics, and Biotechnology at the University of Warmia and Mazury in Olsztyn.

Ten plants were cultured for 14 days in 200 mL glass jars containing 100 mL of a liquid 50% MS medium. The culture conditions were set at a temperature of 23 °C/17 °C (day/night) with a 16/8 h photoperiod and a light intensity of 3.4 klx (Osram L36W/77 Fluora fluorescent lamp by OSRAM Licht AG, Munich, Germany). The medium was supplemented with fluoxetine at concentrations ranging from 0 to 150 mg L^−1^.

**Figure 8:**
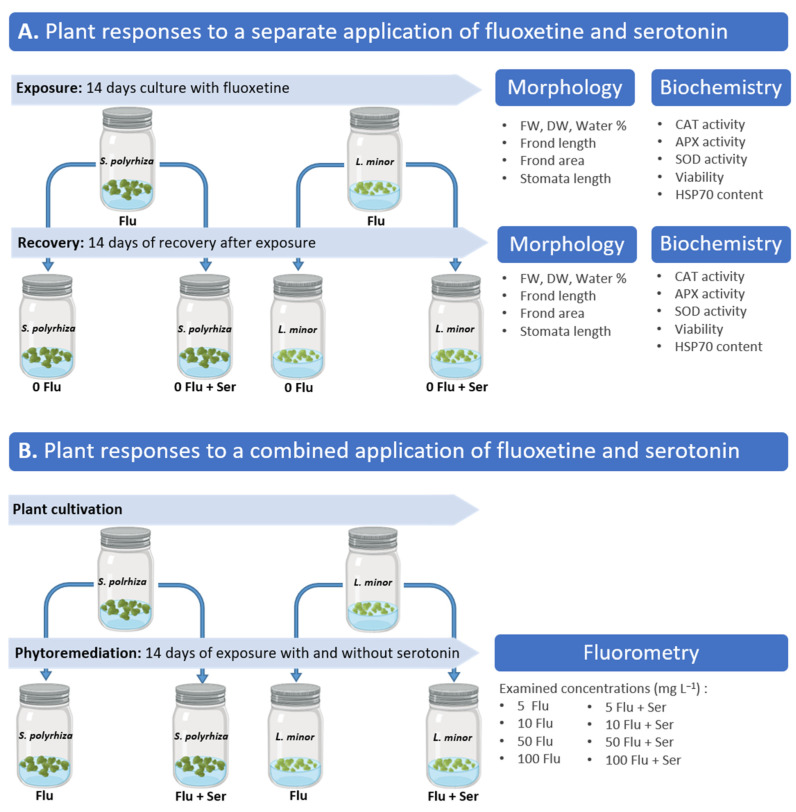
Summary of the experimental design assessing the effects of fluoxetine and Ser on *S. polyrhiza* and *L. minor*. Panel (**A**) shows plant exposure to fluoxetine followed by a recovery phase with or without Ser (100 µM), with assessments of morphological and biochemical parameters. Panel (**B**) presents the combined treatments of fluoxetine and Ser (100 µM), including fluorometric analyses across four fluoxetine concentrations.

After the 14-day exposure period, the plants were transferred to a fresh medium without fluoxetine for a recovery phase. Some plants were transferred to a fresh medium without fluoxetine but supplemented with 100 µM of Ser. Plant responses were analyzed after the initial 14-day fluoxetine exposure and again after the subsequent 14-day recovery phase. A methodological scheme (Figure 8) was prepared to better illustrate the sequence of actions and analyses.

For the experiment testing the phytoremediation capabilities of duckweeds, the plant culture was maintained under the same conditions except for medium supplementation. The experiment lasted 14 days and the culture medium was supplemented with fluoxetine in concentrations of 5, 10, 50, and 100 mg L^−1^ with and without the addition of 100 µM Ser (Figure 8).

### 3.2. Morphological Analyses

The morphological analyses were conducted according to the OECD [58] protocol for *Lemna* sp. Images of the duckweeds were taken using a KEYENCE VHX microscope (Keyence Corporation, Osaka, Japan) (at 20× magnification with a ZS20 objective (Keyence Corporation, Osaka, Japan) for whole plants and 1000× magnification with a ZS200 objective (Keyence Corporation, Osaka, Japan) for stomata).

### 3.3. Enzymatic Activity Measurements

Plant extracts were prepared on ice. The plants were first ground in liquid nitrogen using a porcelain mortar and pestle. For the antioxidant enzymes, the plants were homogenized in 0.05 M K-phosphate buffer (pH 7.0) containing 2% (*w*/*v*) PVPP (polyvinylpolypyrrolidone; Sigma-Aldrich, Poznań, Poland; Cat. No. 77627), 0.4 mM EDTA (ethylenedinitrilotetraacetic acid; aktyn, Poznań, Poland; Cat. No. 607-429-00-8), and 0.2 mM PMSF (phenylmethylsulfonyl fluoride; Roche, Mannheim, Germany; Cat. No. 11359061001) using a Retsch Mixer Mill MM400 (RETSCH GmbH, Haan, Germany). Samples were centrifuged for 20 min at 12,000× *g* at 4 °C. The supernatant was then carefully collected, and the pellet was discarded.

CAT activity was determined spectrophotometrically (Helios Alpha UV-Vis; Thermo Electron Corporation, Waltham, MA, USA) in a reaction mixture containing 50 mM phosphate buffer (pH 7.0) and 15 mM H_2_O_2_ (hydrogen peroxide; Tarchem, Góry Tarnowkie, Poland; Cat. No. 011018). Absorbance was measured at room temperature at 240 nm, according to Aebi [59]. One unit of CAT activity was defined as the reduction of 1 µM of H_2_O_2_ per minute.

APX activity was determined spectrophotometrically in a reaction mixture containing 50 mM phosphate buffer (pH 7.0), 0.5 mM ascorbic acid (Sigma-Aldrich, Poland; Cat. No. 795437), 0.1 mM EDTA, and 0.1 mM H_2_O_2_. Enzyme activity was based on the decrease in absorbance at 290 nm at room temperature, as per Nakano and Asada [60]. APX activity was calculated using an extinction coefficient of 2.8 mM^−1^ cm^−1^. One unit of activity was defined as the amount of enzyme required to oxidize 1 µmol of ascorbate per minute per mg of protein.

SOD activity was assessed by monitoring the inhibition of NBT reduction, following the method of Beauchamp and Fridovich [61]. The reaction mixture contained 50 mM phosphate buffer (pH 7.8), 13 mM methionine (Sigma-Aldrich, Poland; Cat. No. M9625), 63 µM NBT (nitrotetrazolium blue chloride; Sigma-Aldrich, Poland; Cat. No. N6876), 0.1 mM EDTA, and 2 µM riboflavin (Hopkin & Williams, Essex, UK). The mixture was illuminated at an intensity of 50 E m^−2^ s^−1^ for 10 min. Absorbance was measured at 560 nm. One unit of SOD activity was defined as the amount of enzyme that caused a 50% inhibition of NBT reduction.

### 3.4. Cell Viability and Stress Markers

TTC Assay/Viability Test: The reduction of TTC to red formazan was performed according to Steponkus and Lanphear [41]. 0.25 g of plant tissue was incubated in 5 mL of a 1% (*w*/*v*) TTC (2,3,5-triphenyltetrazolium chloride; Sigma-Aldrich, Poland; Cat. No. T8877) solution in phosphate buffer (pH 7.4) at room temperature in the dark. The tissues were then rinsed with distilled water and homogenized using a mortar and pestle in 95% ethanol. The elution of red formazan was performed at 55 °C until the tissue was completely decolorized. After elution, another portion of 95% ethanol was added to bring the final volume to 10 mL. The mixture was then centrifuged for 3 min at 10,000 rcf. The absorbance of the red formazan was measured at 530 nm instead of 485 nm to avoid interference from pigments such as chlorophyll [41].

HSP70 Content: Proteins were isolated using the method of Isaacson et al. [62] with minor modifications. 0.4 g of plant tissue was ground in a cold mortar with 4 mL of 10% TCA (trichloroacetic acid; Sigma-Aldrich, Poland; Cat. No. T4885) in acetone. The extracts were transferred to Eppendorf tubes and incubated at −20 °C for 24 h. The extracts were then centrifuged at 5000× *g* for 30 min. The extracts were purified by adding 4 mL of cold acetone, and this rinsing step was repeated twice. The mixture was then centrifuged for 10 min at 4 °C at 5000× *g*. The pellet was dried at room temperature and then resuspended in TBS buffer containing 250 mM Tris and 1.37 M NaCl. The content of HSP70 was determined using an ELISA kit (EIAab Science, Wuhan, China). 100 µL of protein extract was applied to a 96-well plate and incubated at 37 °C. Subsequent steps were performed according to the manufacturer’s protocol, and the plate was incubated again at 37 °C for one hour. The wells were then rinsed, substrate was added, and the reaction was carried out at 37 °C for 20 min. Absorbance was measured at a wavelength of 450 nm as per the manufacturer’s protocol.

### 3.5. Fluorometric Determination of Fluoxetine in the Medium

The amount of fluoxetine was determined by fluorometric analysis of the generated Flu-NBD fluorophore according to Darwish et al. [63] with minor modifications. The effect of Ser on the elimination of fluoxetine from the medium was also examined. The RFI of the resulting solution was measured at λ_ex_ = 490 nm and λ_em_ = 545 nm relative to a blank sample prepared in the same manner with 1 mL of water instead of 1 mL of fluoxetine solution.

### 3.6. Spectrophotometric Measurements

To determine the changes in the absorption spectrum of the drug and Ser over time, serotonin (serotonin hydrochloride; Sigma-Aldrich, Poland; Cat. No. H9523) (100 µM), fluoxetine (fluoxetine hydrochloride; Sigma-Aldrich, Poland; Cat. No. PHR1394) (100 mg L^−1^), and a mixture of these substances dissolved in water were analyzed in the wavelength range of 250–350 nm. The results of the absorbance measurements for Ser and the substance mixture were normalized to a scale from 0 to 1. Absorbance changes were measured using a Shimadzu UV-1900i spectrophotometer (Shimadzu Corporation, Kyoto, Japan) with a quartz cuvette with a stopper for UV/Vis measurements.

### 3.7. Statistical Analysis

The results were analysed in the Statistica software, version 13.3 (TIBCO Software Inc., Palo Alto, CA, USA) using the ANOVA (one-way and two-way) test. The differences between the trials were analysed using Tukey’s post hoc test at the significance level *p* ≤ 0.05. Absorbance values were compared using the Kruskal–Wallis test to assess differences over time across treatment groups and temperatures.

## 4. Conclusions

*L. minor* and *S. polyrhiza* exhibit a high tolerance to fluoxetine, even at concentrations as high as 100 mg L^−1^. The most sensitive indicators of fluoxetine’s adverse effects on these plants were found to be stomatal aperture size and the activity of antioxidant enzymes, specifically CAT, APX, and SOD. There were no significant differences observed in the sensitivity of *Lemna* and *Spirodela* to fluoxetine, nor in their capacity for its removal from the water.

The decrease in fluoxetine concentration in the growth medium was directly proportional to its initial concentration. Furthermore, the addition of exogenous Ser to the medium (at a concentration of 100 µM) significantly enhanced the plants’ ability to remove fluoxetine, increasing the removal efficiency on average by 42% and 33% for *Lemna* and *Spirodela*, respectively.

## Figures and Tables

**Table 1:** Fluoxetine concentrations in M&S medium after 14 days of *L. minor* and *S. polyrhiza* culture with serotonin (Flu + Ser) and without serotonin (Flu), C_0_—initial concentration, C_f_—final concentration. Values are mean ± SD (*n* = 3). The letters indicate statistically significant differences (*p* < 0.05) within each treatment, as determined by one-way ANOVA followed by Tukey’s post-hoc multiple comparison test.

C_0_ (mg L^−1^)	C_f_ *L. minor* (mg L^−1^)	C_f_ *S. polyrhiza* (mg L^−1^)
5 Flu	4.97 ± 0.02 d	4.86 ± 0.04 d
5 Flu + Ser	3.73 ± 0.23 d	3.55 ± 0.27 d
10 Flu	9.41 ± 0.26 cd	9.45 ± 0.21 d
10 Flu + Ser	3.68 ± 0.55 d	6.46 ± 0.39 d
50 Flu	40.40 ± 3.17 b	43.02 ± 2.30 c
50 Flu + Ser	23.49 ± 5.22 c	31.65 ± 3.62 c
100 Flu	69.14 ± 8.08 a	85.85 ± 5.15 a
100 Flu + Ser	40.24 ± 7.64 b	57.12 ± 7.13 b

## Data Availability

The original contributions presented in this study are included in the article/Supplementary Materials. Further inquiries can be directed to the corresponding author.

## Supplementary Materials

The following supporting information can be downloaded at: https://www.mdpi.com/article/10.3390/ijms27010002/s1.

## Abbreviations

The following abbreviations are used in this manuscript:

Ser	serotonin
Flu	fluoxetine
CAT	catalase
APX	peroxidase
SOD	superoxide dismutase
SSRI	selective serotonin reuptake inhibitor
TTC	2,3,5-triphenyltetrazolium chloride
HSP70	heat-shock proteins
BF	bioconcentration factor
RFI	relative fluorescence intensity
MS	Murashige and Skoog medium
PPCPs	pharmaceuticals and personal care products
EDCs	endocrine-disrupting chemicals
