# Supplementary material for: Serotonin Application Decreases Fluoxetine-Induced Stress in Lemna minor and Spirodela polyrhiza
Source: Int J Mol Sci. 2025 Dec 19;27(1):2. doi: 10.3390/ijms27010002 (PMC12785827; doi:10.3390/ijms27010002)
Supplement: Supplementary file 1 [file ijms-27-00002-s001.zip › ijms-3958563-supplementary.pdf]

Serotonin application decreases fluoxetine-induced stress in *Lemna minor* and *Spirodela polyrhiza*

Marta Wierzbicka, Dariusz J. Michalczyk, Agnieszka I. Piotrowicz-Cieślak
Department of Plant Physiology, Genetics and Biotechnology, University of Warmia and Mazury in Olsztyn, Poland.

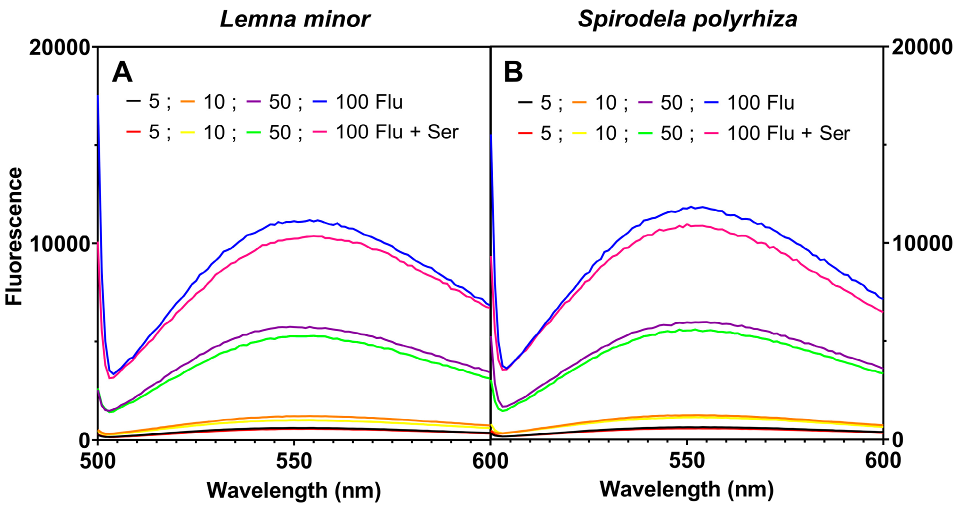

Figure S1. A-B Fluorescence of fluoxetine (Flu) and fluoxetine with the addition/in presence of serotonin (Flu + Ser) at initial concentrations: 5, 10, 50, and 100 mg L^{-1}.

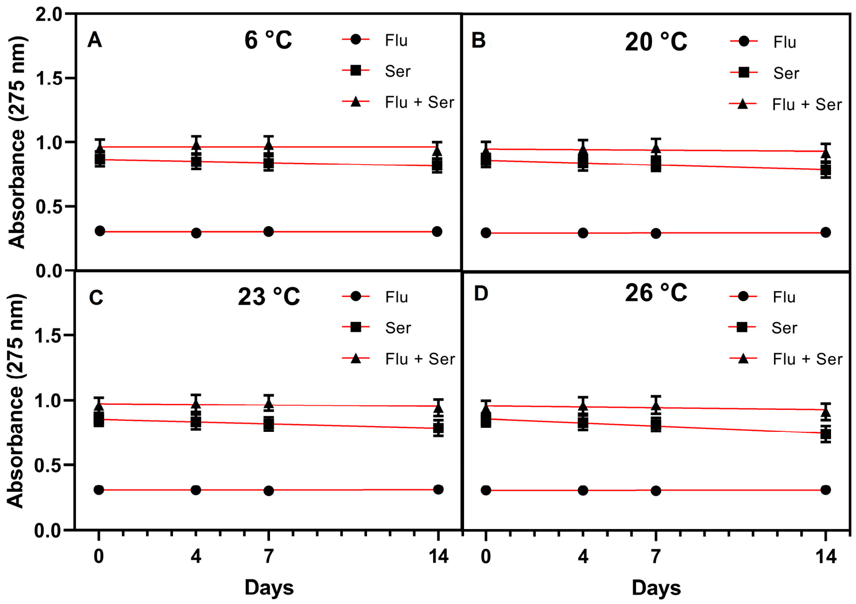

Figure S2. Stability of fluoxetine (Flu), serotonin (Ser), and their mixture (Flu + Ser) stored for 14 days under four temperature regimes (A: 6 °C; B: 20 °C; C: 23 °C; D: 26 °C). Absorbance at 275 nm was monitored on days 0, 4, 7, and 14. Error bars represent standard deviations. Statistical evaluation using the Kruskal–Wallis test revealed no significant differences in absorbance values over time for any treatment group at any temperature.

Table S1.

Fresh weight (FW, mg) of *L. minor*, obtained from 10 plants, measured after fluoxetine exposure (Flu, n=3) and regeneration (from exposure) in two experimental conditions: transfer to medium without fluoxetine (0 Flu, n=3) and transfer to medium supplemented with serotonin (0 Flu + Ser, n=3). Values presented as Mean ± standard deviation (SD) of replicate measurements. The letters indicate statistically significant differences ($p < 0.05$) among fluoxetine concentrations within each treatment, as determined by one-way ANOVA followed by Tukey's post-hoc multiple comparison test.

Fluoxetine concentration (mg L^{-1})	Flu			Flu → 0 Flu			Flu → 0 Flu + Ser		
	Mean	± SD	Group	Mean	± SD	Group	Mean	± SD	Group
0	1309.99	± 65.49	a	1300.66	± 40.77	a	1337.32	± 53.11	A
0.001	1258.22	± 59.17	ab	1292.55	± 42.89	a	1345.55	± 52.05	a
0.005	1308.27	± 59.72	a	1311.27	± 40.57	a	1327.27	± 50.80	a
0.01	1243.71	± 55.19	ab	1265.04	± 37.44	a	1323.38	± 47.33	a
0.05	1246.86	± 59.87	ab	1277.86	± 40.81	a	1310.20	± 48.91	a
0.1	1255.37	± 57.22	ab	1273.70	± 38.45	a	1304.70	± 48.49	a
0.5	1247.86	± 53.25	ab	1272.53	± 32.75	a	1290.53	± 55.13	a
1	1192.54	± 51.50	ab	1275.88	± 33.74	a	1294.21	± 44.73	a
5	1199.28	± 65.58	ab	1301.62	± 37.55	a	1308.62	± 48.69	a
10	1114.80	± 55.62	b	1292.42	± 32.06	a	1286.13	± 44.31	a
50	478.40	± 62.57	c	988.41	± 47.06	b	1086.43	± 45.58	b
100	236.43	± 41.54	d	437.09	± 57.53	c	473.27	± 49.21	c
150	30.93	± 14.62	e	24.26	± 10.10	d	23.26	± 9.383	d

Table S2.

Fresh weight (FW, mg) of *S. polyrhiza*, obtained from 10 plants, measured after fluoxetine exposure (Flu, n=3) and regeneration (from exposure) in two experimental conditions: transfer to medium without fluoxetine (0 Flu, n=3) and transfer to medium supplemented with serotonin (0 Flu + Ser, n=3). Values presented as Mean ± standard deviation (SD) of replicate measurements. The letters indicate statistically significant differences ($p < 0.05$) among fluoxetine concentrations within each treatment, as determined by one-way ANOVA followed by Tukey's post-hoc multiple comparison test.

Fluoxetine concentration (mg L^{-1})	Flu			Flu → 0 Flu			Flu → 0 Flu + Ser		
	Mean	± SD	Group	Mean	± SD	Group	Mean	± SD	Group
0	6260.34	± 193.76	a	6004.95	± 186.18	a	6559.23	± 242.04	a
0.001	6189.15	± 171.06	a	6101.09	± 198.06	a	6447.17	± 212.56	a
0.005	6012.28	± 169.83	ab	6207.45	± 213.14	a	6528.81	± 244.86	a
0.01	6035.54	± 192.41	ab	6180.40	± 230.91	a	6429.93	± 245.94	a
0.05	5926.48	± 165.35	ab	6116.64	± 212.80	a	6482.37	± 204.55	a
0.1	5972.77	± 209.05	ab	6292.74	± 235.45	a	6461.80	± 228.32	a
0.5	5894.55	± 195.36	ab	6070.07	± 183.28	a	6355.44	± 181.88	a
1	5547.54	± 179.43	b	6090.01	± 220.38	a	6326.22	± 209.07	a
5	4793.23	± 195.93	c	5900.73	± 166.35	a	6109.66	± 206.05	a
10	3970.10	± 208.57	d	5712.41	± 231.88	ab	6228.63	± 225.58	a
50	2727.33	± 171.30	e	5104.64	± 255.77	b	5293.72	± 239.22	b
100	750.20	± 204.90	f	3492.92	± 228.03	c	3455.34	± 179.99	c
150	158.67	± 52.01	g	125.10	± 27.84	d	118.00	± 29.58	d

Table S3.

Dry mass (DW, %) of *L. minor* plants measured after fluoxetine exposure (Flu, n=3) and regeneration (from exposure) in two experimental conditions: transfer to medium without fluoxetine (0 Flu, n=3) and transfer to medium supplemented with serotonin (0 Flu + Ser, n=3). Values are presented as Mean ± standard deviation (SD) of replicate measurements. The letters indicate statistically significant differences ($p < 0.05$) among fluoxetine concentrations within each treatment, as determined by one-way ANOVA after logarithmic transformation of the data followed by Tukey's post-hoc multiple comparison test.

Fluoxetine concentration (mg L^{-1})	Flu			Flu → 0 Flu			Flu → 0 Flu + Ser		
	Mean	± SD	Group	Mean	± SD	Group	Mean	± SD	Group
0	8.89	± 0.49	de	8.93	± 0.49	c	9.04	± 0.49	c
0.001	8.80	± 0.50	de	8.91	± 0.50	c	9.16	± 0.50	c
0.005	8.85	± 0.39	de	9.02	± 0.29	c	9.15	± 0.32	c
0.01	8.79	± 0.61	de	9.07	± 0.41	c	9.01	± 0.31	c
0.05	8.73	± 0.51	e	9.08	± 0.51	c	8.92	± 0.51	c
0.1	8.69	± 0.31	e	8.76	± 0.42	c	9.19	± 0.41	c
0.5	8.79	± 0.42	de	8.81	± 0.32	c	8.74	± 0.32	c
1	8.91	± 0.30	de	9.17	± 0.41	c	8.94	± 0.46	c
5	10.02	± 0.45	cd	9.08	± 0.55	c	9.07	± 0.35	c
10	10.20	± 0.39	c	9.00	± 0.39	c	9.06	± 0.39	c
50	10.95	± 0.31	bc	10.01	± 0.54	bc	9.60	± 0.35	c
100	11.90	± 0.29	b	11.04	± 0.31	b	10.92	± 0.31	b
150	23.00	± 0.50	a	24.39	± 0.35	a	23.19	± 0.25	a

Table S4.

Dry mass (DW, %) of *S. polyrhiza* plants measured after fluoxetine exposure (Flu, n=3) and regeneration (from exposure) in two experimental conditions: transfer to medium without fluoxetine (0 Flu, n=3) and transfer to medium supplemented with serotonin (0 Flu + Ser, n=3). Values are presented as Mean ± standard deviation (SD) of replicate measurements. The letters indicate statistically significant differences ($p < 0.05$) among fluoxetine concentrations within each treatment, as determined by one-way ANOVA after logarithmic transformation followed by Tukey's post-hoc multiple comparison test.

Fluoxetine concentration (mg L^{-1})	Flu			Flu → 0 Flu			Flu → 0 Flu + Ser		
	Mean	± SD	Group	Mean	± SD	Group	Mean	± SD	Group
0	8.39	± 0.32	cd	8.37	± 0.49	b	8.56	± 0.49	b
0.001	8.41	± 0.48	cd	8.53	± 0.50	b	8.25	± 0.50	b
0.005	8.30	± 0.31	cd	8.42	± 0.32	b	8.58	± 0.42	b
0.01	8.07	± 0.30	d	8.25	± 0.31	b	8.34	± 0.52	b
0.05	8.12	± 0.25	d	8.69	± 0.51	b	8.43	± 0.51	b
0.1	8.51	± 0.41	cd	8.76	± 0.41	b	8.27	± 0.32	b
0.5	8.50	± 0.40	cd	8.56	± 0.42	b	8.39	± 0.42	b
1	8.18	± 0.34	cd	8.71	± 0.55	b	8.60	± 0.51	b
5	8.60	± 0.30	cd	8.53	± 0.55	b	8.33	± 0.55	b
10	8.41	± 0.20	cd	8.68	± 0.39	b	8.68	± 0.49	b
50	9.20	± 0.36	c	8.87	± 0.41	b	8.70	± 0.54	b
100	10.99	± 0.49	b	9.20	± 0.41	b	9.39	± 0.31	b
150	25.00	± 0.49	a	25.11	± 0.25	a	24.64	± 0.45	a

Table S5.

Water content (Water %) of *L. minor* plants measured after fluoxetine exposure (Flu, n=3) and regeneration (from exposure) in two experimental conditions: transfer to medium without fluoxetine (0 Flu, n=3) and transfer to medium supplemented with serotonin (0 Flu + Ser, n=3). Values presented as Mean ± standard deviation (SD) of replicate measurements. The letters indicate statistically significant differences ($p < 0.05$) among fluoxetine concentrations within each treatment, as determined by one-way ANOVA after logarithmic transformation followed by Tukey's post-hoc multiple comparison test.

Fluoxetine concentration (mg L^{-1})	Flu			Flu → 0 Flu			Flu → 0 Flu + Ser		
	Mean	± SD	Group	Mean	± SD	Group	Mean	± SD	Group
0	91.11	± 0.49	a	91.07	± 0.60	a	90.96	± 0.49	a
0.001	91.10	± 0.52	a	91.09	± 0.55	a	90.84	± 0.42	a
0.005	91.15	± 0.42	a	90.98	± 0.59	a	90.85	± 0.52	a
0.01	91.16	± 0.53	a	90.93	± 0.44	a	90.99	± 0.53	a
0.05	91.27	± 0.45	a	90.92	± 0.51	a	91.08	± 0.51	a
0.1	91.20	± 0.52	a	91.24	± 0.44	a	90.81	± 0.49	a
0.5	91.21	± 0.51	a	91.19	± 0.60	a	91.26	± 0.42	a
1	91.08	± 0.46	a	90.83	± 0.55	a	91.06	± 0.52	a
5	89.98	± 0.55	ab	90.93	± 0.59	a	90.93	± 0.53	a
10	89.19	± 0.59	bc	91.00	± 0.44	a	90.94	± 0.51	a
50	89.05	± 0.44	bc	89.78	± 0.51	ab	90.31	± 0.40	ab
100	88.10	± 0.51	c	88.96	± 0.44	b	89.08	± 0.50	b
150	75.00	± 0.44	d	75.61	± 0.29	c	75.81	± 0.40	c

Table S6.

Water content (Water %) of *S. polyrhiza* plants measured after fluoxetine exposure (Flu, n=3) and regeneration (from exposure) in two experimental conditions: transfer to medium without fluoxetine (0 Flu, n=3) and transfer to medium supplemented with serotonin (0 Flu + Ser, n=3). Values are presented as Mean ± standard deviation (SD) of replicate measurements. The letters indicate statistically significant differences ($p < 0.05$) among fluoxetine concentrations within each treatment, as determined by one-way ANOVA after logarithmic transformation followed by Tukey's post-hoc multiple comparison test.

Fluoxetine concentration (mg L^{-1})	Flu			Flu → 0 Flu			Flu → 0 Flu + Ser		
	Mean	± SD	Group	Mean	± SD	Group	Mean	± SD	Group
0	91.61	± 0.38	a	91.63	± 0.50	a	91.44	± 0.52	a
0.001	91.59	± 0.50	a	91.47	± 0.31	a	91.75	± 0.41	a
0.005	92.00	± 0.31	a	91.58	± 0.37	a	91.42	± 0.50	a
0.01	91.93	± 0.47	a	91.75	± 0.52	a	91.66	± 0.45	a
0.05	91.90	± 0.52	a	91.31	± 0.41	a	91.57	± 0.40	a
0.1	91.50	± 0.41	a	91.24	± 0.50	a	91.73	± 0.52	a
0.5	92.06	± 0.36	a	91.44	± 0.31	a	91.61	± 0.41	a
1	91.82	± 0.44	a	91.29	± 0.27	a	91.40	± 0.50	a
5	91.49	± 0.49	a	91.47	± 0.52	a	91.67	± 0.45	a
10	91.59	± 0.48	a	91.32	± 0.41	a	91.32	± 0.40	a
50	90.70	± 0.51	a	90.97	± 0.50	a	90.80	± 0.48	a
100	89.01	± 0.50	b	90.80	± 0.45	a	90.61	± 0.41	a
150	73.00	± 0.51	c	74.39	± 0.40	b	75.36	± 0.50	b

Table S7.

Frond length (mm) of *L. minor* plants measured after fluoxetine exposure (Flu, n=30) and regeneration (from exposure) in two experimental conditions: transfer to medium without fluoxetine (0 Flu, n=30) and transfer to medium supplemented with serotonin (0 Flu + Ser, n=30). Values are presented as Mean ± standard deviation (SD) of replicate measurements. The letters indicate statistically significant differences ($p < 0.05$) among fluoxetine concentrations within each treatment, as determined by one-way ANOVA followed by Tukey's post-hoc multiple comparison test.

Fluoxetine concentration (mg L^{-1})	Flu			Flu → 0 Flu			Flu → 0 Flu + Ser		
	Mean	± SD	Group	Mean	± SD	Group	Mean	± SD	Group
0	3.76	± 0.25	a	3.79	± 0.24	ab	3.89	± 0.20	a
0.001	3.63	± 0.29	abc	3.76	± 0.26	ab	3.89	± 0.28	a
0.005	3.72	± 0.19	ab	3.82	± 0.20	a	3.91	± 0.27	a
0.01	3.67	± 0.22	ab	3.77	± 0.26	ab	3.92	± 0.22	a
0.05	3.68	± 0.20	ab	3.74	± 0.20	ab	3.90	± 0.22	a
0.1	3.66	± 0.25	ab	3.79	± 0.18	ab	3.83	± 0.23	a
0.5	3.67	± 0.23	ab	3.81	± 0.24	a	3.79	± 0.22	a
1	3.56	± 0.25	bc	3.75	± 0.24	ab	3.80	± 0.30	a
5	3.46	± 0.19	cd	3.71	± 0.24	abc	3.89	± 0.20	a
10	3.53	± 0.17	bc	3.64	± 0.26	abc	3.84	± 0.18	a
50	3.33	± 0.22	de	3.60	± 0.21	bc	3.82	± 0.16	a
100	3.18	± 0.19	e	3.52	± 0.15	c	3.59	± 0.14	b
150	2.90	± 0.16	f	2.78	± 0.16	d	2.68	± 0.19	c

Table S8.

Frond length (mm) of *S. polyrhiza* plants measured after fluoxetine exposure (Flu, n=30) and regeneration (from exposure) in two experimental conditions: transfer to medium without fluoxetine (0 Flu, n=30) and transfer to medium supplemented with serotonin (0 Flu + Ser, n=30). Values are presented as Mean ± standard deviation (SD) of replicate measurements. The letters indicate statistically significant differences ($p < 0.05$) among fluoxetine concentrations within each treatment, as determined by one-way ANOVA followed by Tukey's post-hoc multiple comparison test.

Fluoxetine concentration (mg L^{-1})	Flu			Flu → 0 Flu			Flu → 0 Flu + Ser		
	Mean	± SD	Group	Mean	± SD	Group	Mean	± SD	Group
0	6.76	± 0.20	a	6.81	± 0.13	a	6.85	± 0.12	ab
0.001	6.74	± 0.22	ab	6.78	± 0.13	ab	6.83	± 0.12	ab
0.005	6.64	± 0.21	bc	6.76	± 0.14	ab	6.81	± 0.14	ab
0.01	6.68	± 0.20	bc	6.72	± 0.14	abc	6.87	± 0.13	a
0.05	6.65	± 0.21	bc	6.73	± 0.13	abc	6.84	± 0.13	ab
0.1	6.63	± 0.18	bc	6.70	± 0.13	abc	6.82	± 0.12	ab
0.5	6.62	± 0.15	bc	6.69	± 0.13	bc	6.78	± 0.13	ab
1	6.59	± 0.16	bc	6.70	± 0.14	abc	6.75	± 0.10	b
5	6.56	± 0.16	c	6.69	± 0.13	bc	6.81	± 0.13	ab
10	6.34	± 0.15	d	6.64	± 0.14	c	6.64	± 0.14	cd
50	6.20	± 0.17	d	6.62	± 0.13	c	6.60	± 0.13	de
100	5.94	± 0.16	e	6.39	± 0.12	d	6.52	± 0.14	e
150	5.90	± 0.16	e	5.78	± 0.13	e	5.81	± 0.14	f

Table S9.

Frond area (mm^2) of *L. minor* plants measured after fluoxetine exposure (Flu, n=30) and regeneration (from exposure) in two experimental conditions: transfer to medium without fluoxetine (0 Flu, n=30) and transfer to medium supplemented with serotonin (0 Flu + Ser, n=30). Values are presented as Mean ± standard deviation (SD) of replicate measurements. The letters indicate statistically significant differences ($p < 0.05$) among fluoxetine concentrations within each treatment, as determined by one-way ANOVA followed by Tukey's post-hoc multiple comparison test.

Fluoxetine concentration (mg L^{-1})	Flu			Flu → 0 Flu			Flu → 0 Flu + Ser		
	Mean	± SD	Group	Mean	± SD	Group	Mean	± SD	Group
0	7.91	± 0.36	ab	8.01	± 0.45	a	8.37	± 0.42	a
0.001	7.97	± 0.38	a	7.77	± 0.36	ab	8.37	± 0.46	a
0.005	7.73	± 0.34	abc	7.92	± 0.50	a	8.29	± 0.37	a
0.01	7.90	± 0.33	ab	7.90	± 0.40	a	8.48	± 0.29	a
0.05	7.65	± 0.25	bc	8.05	± 0.36	a	8.40	± 0.34	a
0.1	7.48	± 0.34	c	7.98	± 0.47	a	8.37	± 0.34	a
0.5	7.67	± 0.23	abc	7.89	± 0.48	a	8.31	± 0.26	a
1	7.05	± 0.39	d	8.08	± 0.44	a	8.29	± 0.23	a
5	6.79	± 0.38	d	7.84	± 0.24	ab	8.29	± 0.45	a
10	6.76	± 0.37	d	7.85	± 0.50	ab	8.24	± 0.39	a
50	6.26	± 0.48	e	7.52	± 0.42	b	8.30	± 0.28	a
100	5.54	± 0.36	f	7.11	± 0.48	c	7.30	± 0.38	b
150	4.15	± 0.42	g	3.56	± 0.27	d	3.48	± 0.23	c

Table S10.

Frond area (mm^2) of *S. polyrhiza* plants measured after fluoxetine exposure (Flu, n=30) and regeneration (from exposure) in two experimental conditions: transfer to medium without fluoxetine (0 Flu, n=30) and transfer to medium supplemented with serotonin (0 Flu + Ser, n=30). Values are presented as Mean ± standard deviation (SD) of replicate measurements. The letters indicate statistically significant differences ($p < 0.05$) among fluoxetine concentrations within each treatment, as determined by one-way ANOVA followed by Tukey's post-hoc multiple comparison test.

Fluoxetine concentration (mg L^{-1})	Flu			Flu → 0 Flu			Flu → 0 Flu + Ser		
	Mean	± SD	Group	Mean	± SD	Group	Mean	± SD	Group
0	26.67	± 0.80	a	26.80	± 0.54	a	27.28	± 0.58	ab
0.001	26.69	± 0.75	a	26.72	± 0.58	ab	26.99	± 0.51	bc
0.005	26.54	± 0.76	a	26.86	± 0.58	a	27.38	± 0.56	a
0.01	26.49	± 0.65	a	26.71	± 0.51	ab	26.99	± 0.40	abc
0.05	26.56	± 0.96	a	26.69	± 0.56	ab	27.10	± 0.58	abc
0.1	26.43	± 0.76	ab	26.62	± 0.40	ab	26.83	± 0.51	c
0.5	26.49	± 0.84	a	26.61	± 0.52	ab	26.89	± 0.56	bc
1	25.72	± 0.74	bc	26.63	± 0.55	ab	27.04	± 0.40	abc
5	25.12	± 0.89	cd	26.43	± 0.54	ab	26.91	± 0.52	bc
10	24.91	± 0.91	d	26.31	± 0.56	b	26.85	± 0.55	c
50	23.83	± 0.75	e	26.33	± 0.58	b	26.50	± 0.54	d
100	21.79	± 0.96	f	24.58	± 0.58	c	25.61	± 0.45	e
150	20.11	± 0.85	g	19.91	± 0.29	d	20.19	± 0.27	f

Table S11.

Stomata length (µm) of *L. minor* plants measured after fluoxetine exposure (Flu, n=30) and regeneration (from exposure) in two experimental conditions: transfer to medium without fluoxetine (0 Flu, n=30) and transfer to medium supplemented with serotonin (0 Flu + Ser, n=30). Values presented as Mean ± standard deviation (SD) of replicate measurements. The letters indicate statistically significant differences (p < 0.05) among fluoxetine concentrations within each treatment, as determined by one-way ANOVA followed by Tukey's post-hoc multiple comparison test.

Fluoxetine concentration (mg L^{-1})	Flu			Flu → 0 Flu			Flu → 0 Flu + Ser		
	Mean	± SD	Group	Mean	± SD	Group	Mean	± SD	Group
0	30.82	± 0.68	a	30.65	± 0.60	ab	31.09	± 0.78	a
0.001	30.62	± 0.60	a	30.78	± 0.60	a	31.10	± 0.46	a
0.005	30.55	± 0.47	a	30.80	± 0.43	a	31.09	± 0.47	a
0.01	30.28	± 0.86	ab	30.20	± 0.76	bc	31.07	± 0.69	a
0.05	30.58	± 0.75	a	30.79	± 0.48	a	30.63	± 0.55	abc
0.1	29.75	± 0.60	bc	30.60	± 0.43	ab	30.90	± 0.71	ab
0.5	29.59	± 0.91	c	30.51	± 0.65	abc	30.61	± 0.51	abc
1	29.37	± 0.84	c	30.84	± 0.39	a	30.45	± 0.48	bc
5	29.27	± 0.69	c	30.09	± 0.49	c	30.39	± 0.49	bcd
10	28.41	± 0.65	d	30.41	± 0.53	abc	30.30	± 0.58	cd
50	26.57	± 0.97	e	29.49	± 0.67	d	29.90	± 0.71	d
100	26.54	± 0.98	e	28.67	± 0.72	e	27.97	± 0.66	e
150	24.70	± 0.96	f	24.80	± 0.66	f	24.92	± 0.46	f

Table S12.

Stomata length (µm) of *S. polyrhiza* plants measured after fluoxetine exposure (Flu, n=30) and regeneration (from exposure) in two experimental conditions: transfer to medium without fluoxetine (0 Flu, n=30) and transfer to medium supplemented with serotonin (0 Flu + Ser, n=30). Values presented as Mean ± standard deviation (SD) of replicate measurements. The letters indicate statistically significant differences (p < 0.05) among fluoxetine concentrations within each treatment, as determined by one-way ANOVA after logarithmic transformation of the data followed by Tukey's post-hoc multiple comparison test.

Fluoxetine concentration (mg L^{-1})	Flu			Flu → 0 Flu			Flu → 0 Flu + Ser		
	Mean	± SD	Group	Mean	± SD	Group	Mean	± SD	Group
0	20.84	± 0.52	a	20.99	± 0.43	ab	21.25	± 0.39	a
0.001	20.77	± 0.39	a	21.09	± 0.23	a	20.92	± 0.24	bc
0.005	20.79	± 0.35	a	20.83	± 0.23	abc	21.10	± 0.23	ab
0.01	20.68	± 0.34	a	20.94	± 0.34	abc	21.11	± 0.31	ab
0.05	20.79	± 0.46	a	20.90	± 0.41	abc	20.99	± 0.44	bc
0.1	20.60	± 0.36	a	20.94	± 0.30	abc	20.98	± 0.32	ab
0.5	20.44	± 0.44	ab	20.80	± 0.40	abc	21.09	± 0.45	ab
1	20.30	± 0.29	ab	20.73	± 0.21	bc	20.98	± 0.45	ab
5	20.02	± 0.31	abc	20.70	± 0.45	bc	20.63	± 0.40	c
10	19.57	± 0.38	bcd	20.67	± 0.23	c	20.88	± 0.29	bc
50	19.38	± 0.37	de	20.30	± 0.38	d	20.35	± 0.37	d
100	18.78	± 0.36	e	19.30	± 0.36	e	19.69	± 0.36	e
150	17.29	± 0.33	f	17.69	± 0.23	f	17.69	± 0.24	f

Table S13.

Catalase activity (CAT, U mg protein^{-1}) of *L. minor* plants measured after fluoxetine exposure (Flu, n=3) and regeneration (from exposure) in two experimental conditions: transfer to medium without fluoxetine (0 Flu, n=3) and transfer to medium supplemented with serotonin (0 Flu + Ser, n=3). Values presented as Mean ± standard deviation (SD) of replicate measurements. The letters indicate statistically significant differences ($p < 0.05$) among fluoxetine concentrations within each treatment, as determined by one-way ANOVA after square-root transformation followed by Tukey's post-hoc multiple comparison test.

Fluoxetine concentration (mg L^{-1})	Flu			Flu → 0 Flu			Flu → 0 Flu + Ser		
	Mean	± SD	Group	Mean	± SD	Group	Mean	± SD	Group
0	0.0289	± 0.0056	b	0.0284	± 0.0037	ab	0.0520	± 0.0040	ab
0.001	0.0299	± 0.0039	b	0.0280	± 0.0032	ab	0.0500	± 0.0025	b
0.005	0.0314	± 0.0039	b	0.0280	± 0.0028	ab	0.0519	± 0.0035	ab
0.01	0.0300	± 0.0040	b	0.0289	± 0.0024	ab	0.0491	± 0.0036	b
0.05	0.0304	± 0.0034	b	0.0307	± 0.0026	a	0.0527	± 0.0025	ab
0.1	0.0310	± 0.0040	b	0.0290	± 0.0023	ab	0.0490	± 0.0032	b
0.5	0.0340	± 0.0034	b	0.0281	± 0.0024	ab	0.0519	± 0.0023	ab
1	0.0513	± 0.0044	a	0.0277	± 0.0034	ab	0.0560	± 0.0024	a
5	0.0548	± 0.0037	a	0.0278	± 0.0035	ab	0.0515	± 0.0034	ab
10	0.0481	± 0.0034	a	0.0264	± 0.0025	ab	0.0522	± 0.0028	ab
50	0.0320	± 0.0024	b	0.0245	± 0.0025	b	0.0533	± 0.0025	ab
100	0.0066	± 0.0024	c	0.0187	± 0.0021	c	0.0264	± 0.0023	c
150	0.0012	± 0.0003	c	0.0004	± 0.0001	d	0.0005	± 0.0001	d

Table S14.

Catalase activity (CAT, U mg protein^{-1}) of *S. polyrhiza* plants measured after fluoxetine exposure (Flu, n=3) and regeneration (from exposure) in two experimental conditions: transfer to medium without fluoxetine (0 Flu, n=3) and transfer to medium supplemented with serotonin (0 Flu + Ser, n=3). Values presented as Mean ± standard deviation (SD) of replicate measurements. The letters indicate statistically significant differences ($p < 0.05$) among fluoxetine concentrations within each treatment, as determined by one-way ANOVA after square-root transformation followed by Tukey's post-hoc multiple comparison test.

Fluoxetine concentration (mg L^{-1})	Flu			Flu → 0 Flu			Flu → 0 Flu + Ser		
	Mean	± SD	Group	Mean	± SD	Group	Mean	± SD	Group
0	0.0210	± 0.0025	ef	0.0212	± 0.0030	ab	0.0342	± 0.0031	a
0.001	0.0220	± 0.0018	def	0.0223	± 0.0020	ab	0.0352	± 0.0030	a
0.005	0.0229	± 0.0023	cdef	0.0235	± 0.0015	a	0.0354	± 0.0028	a
0.01	0.0230	± 0.0016	cdef	0.0208	± 0.0024	ab	0.0320	± 0.0026	a
0.05	0.0249	± 0.0028	cdef	0.0207	± 0.0027	ab	0.0315	± 0.0028	a
0.1	0.0259	± 0.0026	cde	0.0218	± 0.0026	ab	0.0310	± 0.0027	a
0.5	0.0297	± 0.0028	bcd	0.0214	± 0.0026	ab	0.0349	± 0.0026	a
1	0.0309	± 0.0017	bc	0.0217	± 0.0033	ab	0.0335	± 0.0026	a
5	0.0364	± 0.0024	ab	0.0215	± 0.0028	ab	0.0317	± 0.0033	a
10	0.0395	± 0.0026	a	0.0217	± 0.0016	ab	0.0323	± 0.0028	a
50	0.0230	± 0.0017	cdef	0.0201	± 0.0024	ab	0.0331	± 0.0025	a
100	0.0166	± 0.0030	f	0.0134	± 0.0034	b	0.0259	± 0.0022	a
150	0.0027	± 0.0013	g	0.0002	± 0.0001	c	0.0003	± 0.0001	b

Table S15.
Ascorbate peroxidase activity (APX, U mg protein^{-1}) of *L. minor* plants measured after fluoxetine exposure (Flu, n=3) and regeneration (from exposure) in two experimental conditions: transfer to medium without fluoxetine (0 Flu, n=3) and transfer to medium supplemented with serotonin (0 Flu + Ser, n=3). Values presented as Mean ± standard deviation (SD) of replicate measurements. The letters indicate statistically significant differences ($p < 0.05$) among fluoxetine concentrations within each treatment, as determined by one-way ANOVA followed by Tukey's post-hoc multiple comparison test.

Fluoxetine concentration (mg L^{-1})	Flu			Flu → 0 Flu			Flu → 0 Flu + Ser		
	**Mean**	**± SD**	**Group**	**Mean**	**± SD**	**Group**	**Mean**	**± SD**	**Group**
0	2.85	± 0.39	bcd	2.70	± 0.40	a	4.38	± 0.25	a
0.001	2.70	± 0.20	bcd	2.65	± 0.50	a	4.47	± 0.27	a
0.005	2.64	± 0.27	bcd	2.39	± 0.29	a	4.36	± 0.29	a
0.01	2.65	± 0.19	bcd	2.45	± 0.31	a	4.51	± 0.23	a
0.05	2.73	± 0.34	bcd	2.75	± 0.30	a	4.20	± 0.19	a
0.1	2.56	± 0.34	cd	2.57	± 0.23	a	4.22	± 0.21	a
0.5	2.39	± 0.34	cd	2.34	± 0.38	a	4.60	± 0.30	a
1	2.95	± 0.31	abc	2.52	± 0.32	a	4.15	± 0.23	a
5	3.34	± 0.29	abc	2.50	± 0.27	a	4.55	± 0.29	a
10	3.65	± 0.31	ab	2.63	± 0.28	a	4.45	± 0.31	a
50	3.98	± 0.34	a	2.57	± 0.33	a	4.56	± 0.31	a
100	1.78	± 0.27	d	2.04	± 0.19	a	3.91	± 0.27	a
150	0.22	± 0.20	e	0.26	± 0.26	b	0.28	± 0.16	b

Table S16.
Ascorbate peroxidase activity (APX, U mg protein^{-1}) of *S. polyrhiza* plants measured after fluoxetine exposure (Flu, n=3) and regeneration (from exposure) in two experimental conditions: transfer to medium without fluoxetine (0 Flu, n=3) and transfer to medium supplemented with serotonin (0 Flu + Ser, n=3). Values presented as Mean ± standard deviation (SD) of replicate measurements. The letters indicate statistically significant differences ($p < 0.05$) among fluoxetine concentrations within each treatment, as determined by one-way ANOVA after logarithmic transformation of the data followed by Tukey's post-hoc multiple comparison test.

Fluoxetine concentration (mg L^{-1})	Flu			Flu → 0 Flu			Flu → 0 Flu + Ser		
	**Mean**	**± SD**	**Group**	**Mean**	**± SD**	**Group**	**Mean**	**± SD**	**Group**
0	1.98	± 0.15	c	2.00	± 0.14	a	2.90	± 0.13	ab
0.001	2.06	± 0.12	bc	1.85	± 0.20	ab	2.86	± 0.20	ab
0.005	2.07	± 0.20	bc	1.94	± 0.14	ab	2.80	± 0.19	ab
0.01	2.01	± 0.12	c	1.89	± 0.14	ab	2.85	± 0.13	ab
0.05	2.01	± 0.21	c	1.86	± 0.16	ab	3.09	± 0.13	a
0.1	2.18	± 0.13	bc	1.68	± 0.16	ab	2.91	± 0.17	ab
0.5	2.66	± 0.17	ab	1.54	± 0.21	ab	2.94	± 0.14	ab
1	2.87	± 0.14	a	1.77	± 0.19	ab	2.58	± 0.21	ab
5	2.94	± 0.14	a	1.62	± 0.14	ab	2.69	± 0.13	ab
10	2.87	± 0.12	a	1.62	± 0.11	ab	2.53	± 0.14	b
50	2.16	± 0.12	bc	1.72	± 0.13	ab	2.50	± 0.13	b
100	1.10	± 0.11	d	1.40	± 0.12	b	1.92	± 0.15	c
150	0.09	± 0.04	e	0.02	± 0.01	c	0.05	± 0.02	d

Table S17.

Superoxide dismutase activity (SOD, U mg protein-1) of *L. minor* plants measured after fluoxetine exposure (Flu, n=3) and regeneration (from exposure) in two experimental conditions: transfer to medium without fluoxetine (0 Flu, n=3) and transfer to medium supplemented with serotonin (0 Flu + Ser, n=3). Values presented as Mean ± standard deviation (SD) of replicate measurements. The letters indicate statistically significant differences ($p < 0.05$) among fluoxetine concentrations within each treatment, as determined by one-way ANOVA after logarithmic transformation of the data followed by Tukey's post-hoc multiple comparison test.

Fluoxetine concentration (mg L^{-1})	Flu			Flu → 0 Flu			Flu → 0 Flu + Ser		
	Mean	± SD	Group	Mean	± SD	Group	Mean	± SD	Group
0	115.21	± 8.49	ab	118.46	± 9.61	a	141.46	± 10.20	a
0.001	113.46	± 6.64	ab	117.91	± 8.84	a	140.91	± 10.13	a
0.005	113.76	± 7.03	ab	121.27	± 6.05	a	143.27	± 11.07	a
0.01	123.20	± 6.98	a	118.54	± 8.12	a	139.74	± 9.75	a
0.05	125.71	± 7.37	a	113.24	± 7.02	a	140.24	± 11.65	a
0.1	127.60	± 5.63	a	117.03	± 6.65	a	133.03	± 9.02	a
0.5	129.05	± 6.24	a	110.30	± 8.05	a	150.30	± 9.49	a
1	137.16	± 5.01	a	108.10	± 5.09	a	145.10	± 9.93	a
5	120.28	± 6.55	a	118.73	± 8.11	a	142.73	± 8.10	a
10	102.32	± 7.12	ab	115.54	± 6.93	a	145.94	± 10.61	a
50	68.07	± 6.58	b	111.08	± 6.13	a	146.08	± 10.84	a
100	31.11	± 5.30	c	100.04	± 5.10	a	135.04	± 6.05	a
150	9.60	± 4.51	d	5.95	± 2.20	b	3.95	± 2.24	b

Table S18.

Superoxide dismutase activity (SOD, U mg protein-1) of *S. polyrhiza* plants measured after fluoxetine exposure (Flu, n=3) and regeneration (from exposure) in two experimental conditions: transfer to medium without fluoxetine (0 Flu, n=3) and transfer to medium supplemented with serotonin (0 Flu + Ser, n=3). Values presented as Mean ± standard deviation (SD) of replicate measurements. The letters indicate statistically significant differences ($p < 0.05$) among fluoxetine concentrations within each treatment, as determined by one-way ANOVA followed by Tukey's post-hoc multiple comparison test.

Fluoxetine concentration (mg L^{-1})	Flu			Flu → 0 Flu			Flu → 0 Flu + Ser		
	Mean	± SD	Group	Mean	± SD	Group	Mean	± SD	Group
0	105.00	± 6.49	a	107.00	± 8.61	a	135.95	± 8.24	a
0.001	105.21	± 5.06	a	108.91	± 7.84	a	137.14	± 7.16	a
0.005	103.52	± 4.03	a	108.93	± 5.05	a	132.92	± 7.71	a
0.01	105.29	± 6.10	a	106.51	± 5.24	a	138.99	± 9.34	a
0.05	108.76	± 6.04	a	108.45	± 7.02	a	135.65	± 8.13	a
0.1	107.56	± 6.63	a	107.98	± 7.65	a	138.00	± 6.82	a
0.5	116.30	± 6.54	a	105.46	± 7.75	a	135.90	± 9.13	a
1	121.23	± 4.54	a	106.46	± 4.87	a	132.00	± 8.98	a
5	91.49	± 5.95	a	107.45	± 8.71	a	128.00	± 6.00	a
10	86.00	± 5.74	a	102.49	± 4.93	a	132.00	± 8.12	a
50	75.50	± 5.10	a	95.59	± 6.13	ab	137.14	± 8.84	a
100	36.66	± 5.06	b	65.59	± 6.10	b	89.00	± 10.05	b
150	9.92	± 4.71	c	8.95	± 2.80	c	10.95	± 2.24	c

Table S19.

TTC assay (formazan absorbance, nm) of *L. minor* plants measured after fluoxetine exposure (Flu, n=3) and regeneration (from exposure) in two experimental conditions: transfer to medium without fluoxetine (0 Flu, n=3) and transfer to medium supplemented with serotonin (0 Flu + Ser, n=3). Values presented as Mean ± standard deviation (SD) of replicate measurements. The letters indicate statistically significant differences ($p < 0.05$) among fluoxetine concentrations within each treatment, as determined by one-way ANOVA after logarithmic transformation of the data followed by Tukey's post-hoc multiple comparison test.

Fluoxetine concentration (mg L^{-1})	Flu			Flu → 0 Flu			Flu → 0 Flu + Ser		
	Mean	± SD	Group	Mean	± SD	Group	Mean	± SD	Group
0	0.50	± 0.02	a	0.49	± 0.01	a	0.53	± 0.01	a
0.001	0.48	± 0.02	a	0.49	± 0.01	a	0.53	± 0.02	a
0.005	0.48	± 0.02	a	0.50	± 0.02	a	0.53	± 0.02	a
0.01	0.47	± 0.02	a	0.50	± 0.02	a	0.51	± 0.02	a
0.05	0.46	± 0.02	ab	0.49	± 0.01	a	0.50	± 0.02	a
0.1	0.47	± 0.02	a	0.48	± 0.03	a	0.51	± 0.02	a
0.5	0.45	± 0.02	ab	0.48	± 0.01	ab	0.51	± 0.02	a
1	0.45	± 0.02	ab	0.47	± 0.01	ab	0.51	± 0.02	a
5	0.43	± 0.03	ab	0.47	± 0.01	ab	0.49	± 0.02	a
10	0.39	± 0.02	bc	0.46	± 0.02	ab	0.49	± 0.01	a
50	0.32	± 0.03	c	0.42	± 0.02	b	0.48	± 0.01	a
100	0.16	± 0.02	d	0.30	± 0.01	c	0.39	± 0.02	b
150	0.03	± 0.01	e	0.02	± 0.01	d	0.02	± 0.01	c

Table S20.

TTC assay (formazan absorbance, nm) of *S. polyrhiza* plants measured after fluoxetine exposure (Flu, n=3) and regeneration (from exposure) in two experimental conditions transfer to medium without fluoxetine (0 Flu, n=3) and transfer to medium supplemented with serotonin (0 Flu + Ser, n=3). Values presented as Mean ± standard deviation (SD) of replicate measurements. The letters indicate statistically significant differences ($p < 0.05$) among fluoxetine concentrations within each treatment, as determined by one-way ANOVA after logarithmic transformation of the data followed by Tukey's post-hoc multiple comparison test.

Fluoxetine concentration (mg L^{-1})	Flu			Flu → 0 Flu			Flu → 0 Flu + Ser		
	Mean	± SD	Group	Mean	± SD	Group	Mean	± SD	Group
0	0.49	± 0.03	a	0.51	± 0.01	a	0.54	± 0.01	a
0.001	0.49	± 0.02	ab	0.50	± 0.01	ab	0.54	± 0.02	a
0.005	0.50	± 0.02	a	0.50	± 0.01	a	0.52	± 0.01	ab
0.01	0.48	± 0.02	ab	0.50	± 0.02	ab	0.53	± 0.02	a
0.05	0.48	± 0.02	ab	0.47	± 0.02	ab	0.51	± 0.01	ab
0.1	0.47	± 0.01	ab	0.48	± 0.01	ab	0.50	± 0.02	ab
0.5	0.47	± 0.02	ab	0.49	± 0.01	ab	0.51	± 0.02	ab
1	0.47	± 0.02	ab	0.48	± 0.02	ab	0.51	± 0.01	ab
5	0.43	± 0.02	ab	0.48	± 0.02	ab	0.51	± 0.01	ab
10	0.41	± 0.03	b	0.47	± 0.02	ab	0.49	± 0.02	ab
50	0.25	± 0.02	c	0.44	± 0.01	b	0.48	± 0.01	b
100	0.16	± 0.03	d	0.22	± 0.02	c	0.26	± 0.01	c
150	0.03	± 0.01	e	0.02	± 0.01	d	0.03	± 0.01	d

Table S21.

HSP 70 content (ng mL^{-1}) of *L. minor* plants measured after fluoxetine exposure (Flu, n=3) and regeneration (from exposure) in two experimental conditions: transfer to medium without fluoxetine (0 Flu, n=3) and transfer to medium supplemented with serotonin (0 Flu + Ser, n=3). Values presented as Mean ± standard deviation (SD) of replicate measurements. The letters indicate statistically significant differences ($p < 0.05$) among fluoxetine concentrations within each treatment, as determined by one-way ANOVA after logarithmic transformation of the data followed by Tukey's post-hoc multiple comparison test.

Fluoxetine concentration (mg L^{-1})	Flu			Flu → 0 Flu			Flu → 0 Flu + Ser		
	Mean	± SD	Group	Mean	± SD	Group	Mean	± SD	Group
0	2.99	± 0.07	c	2.81	± 0.04	c	3.92	± 0.10	a
0.001	2.98	± 0.05	c	2.99	± 0.04	abc	3.90	± 0.06	a
0.005	2.99	± 0.06	c	2.97	± 0.05	abc	3.95	± 0.10	a
0.01	2.97	± 0.06	c	2.96	± 0.04	bc	3.95	± 0.09	a
0.05	2.94	± 0.08	c	3.06	± 0.05	ab	4.06	± 0.10	a
0.1	2.99	± 0.05	c	3.06	± 0.04	ab	3.92	± 0.06	a
0.5	2.98	± 0.06	c	3.07	± 0.04	ab	3.96	± 0.07	a
1	2.96	± 0.07	c	3.09	± 0.05	ab	3.94	± 0.11	a
5	2.99	± 0.07	c	3.14	± 0.04	ab	4.08	± 0.11	a
10	3.13	± 0.07	bc	3.17	± 0.08	ab	3.94	± 0.08	a
50	3.52	± 0.09	a	3.18	± 0.08	a	4.08	± 0.09	a
100	3.36	± 0.11	ab	3.03	± 0.09	ab	3.80	± 0.08	a
150	1.90	± 0.11	d	1.02	± 0.04	d	0.82	± 0.06	b

Table S22.

HSP 70 content (ng mL^{-1}) of *S. polyrhiza* plants measured after fluoxetine exposure (Flu, n=3) and regeneration (from exposure) in two experimental conditions transfer to medium without fluoxetine (0 Flu, n=3) and transfer to medium supplemented with serotonin (0 Flu + Ser, n=3). Values presented as Mean ± standard deviation (SD) of replicate measurements. The letters indicate statistically significant differences ($p < 0.05$) among fluoxetine concentrations within each treatment, as determined by one-way ANOVA after logarithmic transformation of the data followed by Tukey's post-hoc multiple comparison test.

Fluoxetine concentration (mg L^{-1})	Flu			Flu → 0 Flu			Flu → 0 Flu + Ser		
	Mean	± SD	Group	Mean	± SD	Group	Mean	± SD	Group
0	3.72	± 0.13	cd	3.65	± 0.10	a	4.65	± 0.10	ab
0.001	3.72	± 0.10	cd	3.72	± 0.08	a	4.64	± 0.10	ab
0.005	3.70	± 0.13	cd	3.70	± 0.09	a	4.51	± 0.10	b
0.01	3.73	± 0.09	cd	3.70	± 0.10	a	4.59	± 0.07	ab
0.05	3.71	± 0.12	cd	3.75	± 0.09	a	4.71	± 0.12	ab
0.1	3.61	± 0.10	d	3.76	± 0.10	a	4.62	± 0.10	ab
0.5	3.63	± 0.10	d	3.72	± 0.10	a	4.64	± 0.10	ab
1	3.56	± 0.15	d	3.71	± 0.09	a	4.59	± 0.13	ab
5	4.77	± 0.15	ab	3.62	± 0.11	a	4.89	± 0.11	ab
10	5.07	± 0.18	a	3.56	± 0.14	ab	4.91	± 0.09	a
50	4.19	± 0.15	bc	3.59	± 0.06	ab	4.67	± 0.11	ab
100	3.51	± 0.13	d	3.26	± 0.06	b	4.57	± 0.10	ab
150	1.50	± 0.14	e	1.12	± 0.05	c	1.06	± 0.06	c
